# Antibodies Can Last for More Than 1 Year After SARS-CoV-2 Infection: A Follow-Up Study From Survivors of COVID-19

**DOI:** 10.3389/fmed.2021.684864

**Published:** 2021-07-16

**Authors:** Kaihu Xiao, Haiyan Yang, Bin Liu, Xiaohua Pang, Jianlin Du, Mengqi Liu, Yajie Liu, Xiaodong Jing, Jing Chen, Songbai Deng, Zheng Zhou, Jun Du, Li Yin, Yuling Yan, Huaming Mou, Qiang She

**Affiliations:** ^1^Department of Cardiology, The Second Affiliated Hospital of Chongqing Medical University, Chongqing, China; ^2^Department of Cardiology, Chongqing University Three Gorges Hospital, Chongqing, China; ^3^Department of Geriatrics, Chongqing General Hospital, Chongqing, China; ^4^Department of Radiology, The First Affiliated Hospital of Chongqing Medical University, Chongqing, China; ^5^Department of Medical Laboratory, Chongqing University Three Gorges Hospital, Chongqing, China

**Keywords:** COVID-19, SARS-CoV-2, follow-up, antibody, cardiopulmonary

## Abstract

**Background:** COVID-19 is a global pandemic. The prevention of SARS-CoV-2 infection and the rehabilitation of survivors are currently the most urgent tasks. However, after patients with COVID-19 are discharged from the hospital, how long the antibodies persist, whether the lung lesions can be completely absorbed, and whether cardiopulmonary abnormalities exist remain unclear.

**Methods:** A total of 56 COVID-19 survivors were followed up for 12 months, with examinations including serum virus-specific antibodies, chest CT, and cardiopulmonary exercise testing.

**Results:** The IgG titer of the COVID-19 survivors decreased gradually, especially in the first 6 months after discharge. At 6 and 12 months after discharge, the IgG titer decreased by 68.9 and 86.0%, respectively. The IgG titer in patients with severe disease was higher than that in patients with non-severe disease at each time point, but the difference did not reach statistical significance. Among the patients, 11.8% were IgG negative up to 12 months after discharge. Chest CT scans showed that at 3 and 10 months after discharge, the lung opacity had decreased by 91.9 and 95.5%, respectively, as compared with that at admission. 10 months after discharge, 12.5% of the patients had an opacity percentage >1%, and 18.8% of patients had pulmonary fibrosis (38.5% in the severe group and 5.3% in the non-severe group, *P* < 0.001). Cardiopulmonary exercise testing showed that 22.9% of patients had FEV1/FVC%Pred <92%, 17.1% of patients had FEV1%Pred <80%, 20.0% of patients had a VO_2_ AT <14 mlO_2_/kg/min, and 22.9% of patients had a VE/VCO_2_ slope >30%.

**Conclusions:** IgG antibodies in most patients with COVID-19 can last for at least 12 months after discharge. The IgG titers decreased significantly in the first 6 months and remained stable in the following 6 months. The lung lesions of most patients with COVID-19 can be absorbed without sequelae, and a few patients in severe condition are more likely to develop pulmonary fibrosis. Approximately one-fifth of the patients had cardiopulmonary dysfunction 6 months after discharge.

## Introduction

Coronavirus Disease 2019 (COVID-19), caused by SARS-CoV-2 infection, is still a worldwide pandemic. Globally, as of March 29, 2021, the WHO had reported 126.6 million confirmed cases of COVID-19, including 2.7 million deaths (1). The mortality rate of the disease is ~2%. The prevention of SARS-CoV-2 infection and the rehabilitation of survivors are currently the most urgent tasks. As vaccination becomes widely available and used, the devastating effects of many infectious diseases have faded (2). At present, many countries and regions have begun to vaccinate against SARS-CoV-2 infection. Although how long antibodies last is unclear, this matter is receiving close attention worldwide. SARS-CoV-2 predominantly infects the airways, causing symptoms and disease ranging from mild respiratory infections to severe acute respiratory syndrome, the latter of which results in organ failure in some patients and eventually leads to death (3). Whether the lung lesions of COVID-19 survivors can completely recover is unclear. SARS-CoV-2 infects the human body through binding the transmembrane angiotensin-converting enzyme 2 (ACE2), which is ubiquitously expressed in the nasal epithelium, lung, heart, kidney, and intestines (4). Therefore, COVID-19 also affects multiple organs, particularly the cardiovascular system, and causes arrhythmia and cardiac injury (5). Whether SARS-CoV-2 infection will have sequelae of cardiopulmonary insufficiency remains unknown. The lack of these data makes vaccination and the recovery of survivors more difficult. Therefore, we conducted a 12-month follow-up study on discharged patients with COVID-19, including antibodies, chest CT, and cardiopulmonary function, with the aim of providing more evidence for the rehabilitation of patients and the application of vaccines.

## Methods

### Study Participants and Groups

Study participants: a total of 56 patients with confirmed SARS-CoV-2 infection in Wanzhou District who were admitted to the Chongqing University Three Gorges Hospital between January 23 and March 11 of 2020 were included in the study (Wanzhou District, bordering on Hubei Province, is the hardest-hit area after Hubei Province). Confirmed COVID-19 diagnosis was defined as positivity in a SARS-CoV-2 nucleic acid test performed with the nasopharyngeal swab-PCR method, accompanied by the presence of associated clinical manifestations and lung CT changes. The patients' epidemiological data, demographics (age and sex), contact history and exposure history, and past medical history were collected. The general information on the patients is shown in Table 1.

**Table 1:** Baseline clinical data.

		**Total**	**Non-severe**	**Severe**
Number		56	36	20
Gender, *n* (%)
	Male	28 (50.0%)	17 (47.2%)	11 (55.0%)
	Female	28 (50.0%)	19 (52.8%)	9 (45.0%)
Age, mean (SD)		48 (15)	43 (13)	58 (15)
Length of hospital stay, days,mean (SD)		18.3 (7.7)	17.5 (7.3)	19.8 (8.4)
Time from discharge to follow-up, days, mean (SD)		377.0 (8.7)	377.3 (8.6)	376.3 (9.1)
Comorbidities, *n* (%)
	Hypertension	5 (8.9%)	3 (8.3%)	2 (10.0%)
	Coronary heart disease	2 (3.6%)	1 (2.8%)	1 (5.0%)
	Diabetes	5 (8.9%)	0	5 (25.0%)
	Pulmonary tuberculosis	1 (1.8%)	1 (2.8%)	0
	Asthma	1 (1.8%)	0	1 (5.0%)
	Chronic bronchitis	1 (1.8%)	0	1 (5.0%)

*Data are expressed as n (%) of participants, unless otherwise indicated*.

Patient groups: according to the Novel Coronavirus Pneumonia Diagnosis and Treatment Plan (Provisional 4th Edition) issued by the National Health Commission of the People's Republic of China, patients were diagnosed as having moderate, severe, or critical cases (6). Moderate-type cases had fever and respiratory tract symptoms, and imaging showed lung inflammation signs. Severe-type cases had any of the following: (1) shortness of breath, RR (respiratory rate) ≥ 30 breaths/min; (2) oxygen saturation ≤ 93% at rest; or (3) arterial oxygen partial pressure (PaO_2_)/fraction of inspired oxygen (FiO_2_) ≤ 300 mmHg (1 mmHg = 0.133 kPa). Critical-type patients had any of the following: (1) respiratory failure and a need for mechanical ventilation; (2) shock; or (3) complication of failure of other organs and a need for intensive care unit (ICU) treatment. All 56 patients signed informed consent documents. The patients were followed up from January 23, 2020, to March 5, 2021. The follow-up time was longer than 12 months. There were 36 moderate cases, 16 severe cases, and four critical cases. The patients were further divided into two groups: a severe group (including severe and critical cases) and a non-severe group (including moderate cases). A comparison among groups is shown in Table 1.

This study was approved by the Medical Ethical Committee of The Second Affiliated Hospital of Chongqing Medical University (reference no. 2020-530). Written informed consent was obtained from each enrolled patient.

### Data Collection

The patients received a viral antibody IgG and IgM test every month after discharge, and reexamination of chest CT was performed at 1, 3, 6, and 10 months after discharge (chest CT images at admission and discharge were also collected). Cardiopulmonary exercise testing (CPET) was performed at 6 months after discharge. After CPET, the patients were followed up for a further 6 months to verify the safety of CPET and evaluate whether viral transmission occurred. The IgG/IgM titer detection method was used on fasting serum samples collected from the patients. Antibody testing was performed with an IgG/IgM Antibody Detection Kit (magnetic particle chemiluminescence method) for Novel Coronavirus (2019-nCoV), which was purchased from BioScience (Chongqing) Biotechnology Co., Ltd. Antibody titers were determined on the basis of the sample luminescence value/cutoff (S/CO), and S/CO ≥1.0 was considered positive.

Chest CT: all patients were imaged with a 16-row multidetector CT scanner (Siemens Somatom Sensation; Siemens, Erlangen, Germany) with the following parameters: 120 kVp, 150 mA, 1.5 mm collimation, reconstruction matrix of 512 ×512, and slice thickness of 1.0 mm. The scanning range included the entire chest from the first ribs to the diaphragm. Artificial intelligence software (CT Pneumonia Analysis, Siemens Healthineers, Siemens, Erlangen, Germany) was used to automatically identify and quantify the hyperdense areas of the lung, and the volume of opacity and percentage of opacity were subsequently calculated. Pulmonary fibrosis was defined as architectural distortion on chest CT.

CPET: According to the guidelines of the American College of Cardiology and American Heart Association (ACC/AHA), (7) CPET was performed and supervised by doctors and rehabilitation therapists. (1) Resting lung function test: The test was started 1 h after a meal. Patients rested for 20 min before the test. A Quark CPET system (COSMED, Roma, Italy) was used for the test. (2) After the lung function test was completed, CPET was performed after a 20-min rest. The workload was selected according to patient height, weight, and daily activity capacity, usually 10–15 W/min. To test electrocardiogram (ECG), blood pressure, and oxygen saturation, the patients rode a bicycle while wearing a mask and connected to a monitor. The patients first rested for 3 min, then warmed up for 3 min without a workload. When starting the exercise phase, a ramp protocol was used. The speed was maintained at 60–65 revolutions/min. The exercise time was generally 6–12 min. Patients were asked to exert their maximum effort. During exercise, the blood pressure, SPO_2_, and ECG were closely monitored. If patients felt difficulty breathing, chest tightness, or pain, if the ECG showed an ST-T change indicating myocardial hypoxia, or if the patient could not tolerate the exercise, we considered the workload to have reached the maximum limit. The workload was decreased, and the exercise was continued for another 2–3 min to enter the recovery phase. Patients were observed for 5 min after the test was finished.

### Statistical Analysis

Statistical analyses were performed in SPSS version 20.0 software (SPSS, Chicago, IL, USA). Data are expressed as the mean ± standard deviation or median (interquartile range). Differences between categorical variables were evaluated with Fisher's exact test. Differences between continuous variables were evaluated with *t* test and Kruskal–Wallis H test. For each test, a two-tailed *P*-value < 0.05 was considered significant.

## Results

### General Patient Information

A total of 56 patients were enrolled in this study (Table 1), including 28 men and 28 women. The average age was 48 ± 15 years, and the patients in the severe group (58 ± 15) were significantly older than those in the non-severe group (43 ± 13) (*P* = 0.001). Regarding complications, five cases had hypertension, five cases had diabetes, two cases had coronary heart disease, one case had chronic bronchitis, one case had bronchial asthma, and one case had a history of tuberculosis. From the discharge of the patients to March 5, 2021, the follow-up time was 377 (±8.7) days.

### Antibody Detection

**Figure 1:**
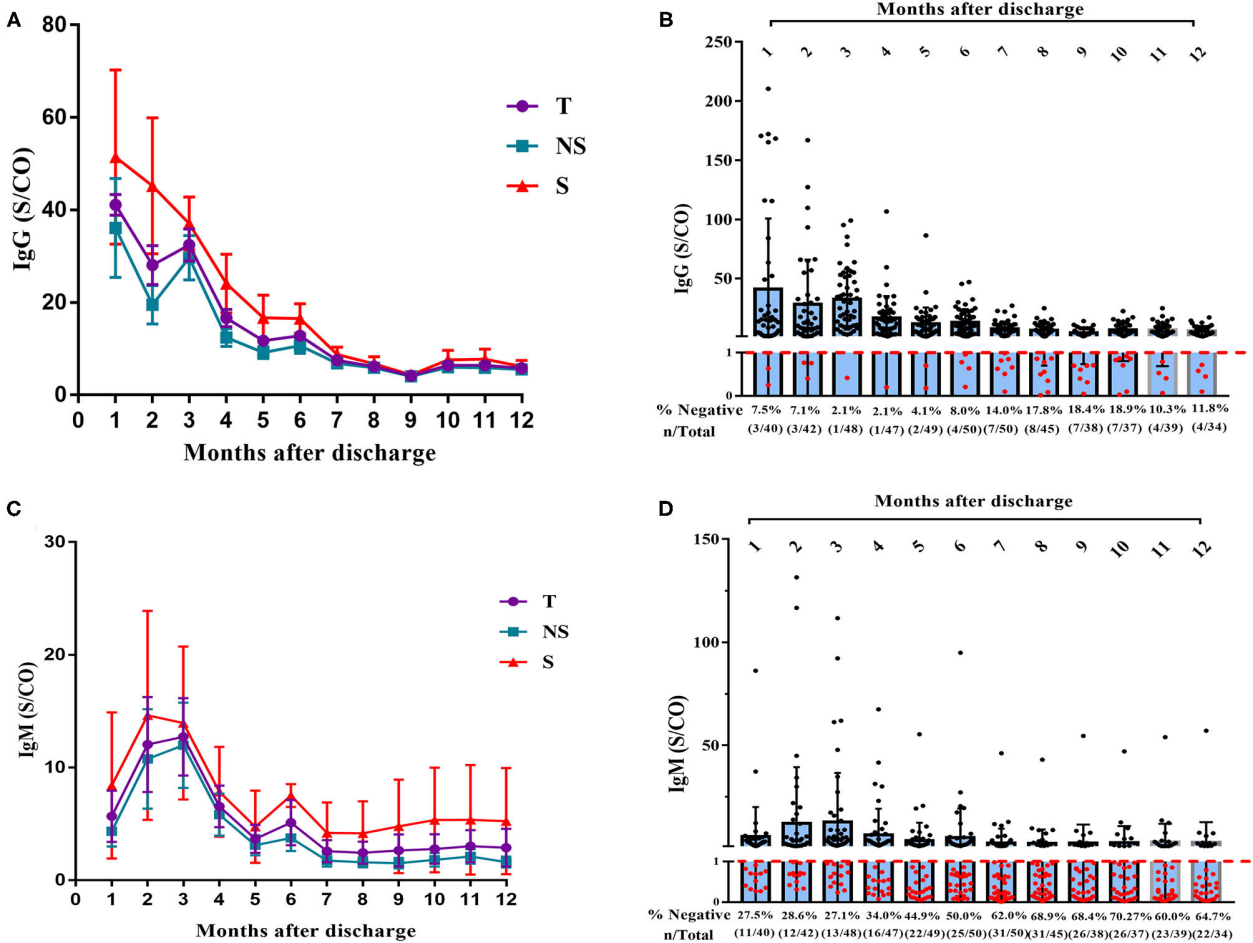
Dynamic changes of IgG and IgM titer over time in 51 patients with COVID-19. **(A)** Dynamic changes of IgG levels in patients with COVID-19 after 12 months' follow-up. **(B)** Negative rate of IgG in COVID-19 patients at each time point with 12 months' follow-up **(C)** Dynamic changes of IgM levels in patients with COVID after 12 months' follow-up. **(D)** Negative rate of IgM in COVID-19 patients at each time point with 12 months' follow-up. T, total; NS, non-severe; S, severe.

We detected patients' serum IgG and IgM to investigate the changes in titers of antibodies to the novel coronavirus. Five patients were lost to follow-up for antibody detection, and 51 patients (18 in the severe group and 33 in the non-severe group) were included in the analysis. As shown in Figure 1, the overall IgG titers gradually decreased, particularly in the first 6 months after discharge. The IgG titer remained stable over 6–12 months, but the level was relatively low (Figure 1A). The IgG titer in patients with a severe disease was higher than that in patients with non-severe disease at each time point, but the difference did not reach statistical significance. At 6 months after discharge, the IgG titer decreased by 68.9% relative to the peak value (70.5% in the non-severe group and 67.9% in the severe group). At 12 months after discharge (13 months after symptom onset), the IgG titer had decreased by 86.0% (84.7% in the non-severe group and 88.1% in the severe group). A total of 8.0% (4/50) of patients were IgG negative at 6 months after discharge, and 11.8% (4/34) of patients were IgG negative at 12 months after discharge (Figure 1B). One patient in the non-severe group was IgG negative during the entire follow-up period. Moreover, the overall trend in IgM showed an initial increase, a peak in the 3rd month after discharge, and then a gradual decrease (Figure 1C). The IgM titer decreased by an average of 59.8% at 6 months after discharge (68.6% in the non-severe group and 48.7% in the severe group) and decreased by an average of 77.2% at 12 months after discharge (86.5% in the non-severe group and 64.2% in the severe group). At 6 months after discharge, 50.0% (25/50) of patients were IgM negative, and at 12 months, 64.7% (22/34) of patients were IgM negative (Figure 1D).

### Radiologic Findings

**Figure 2:**
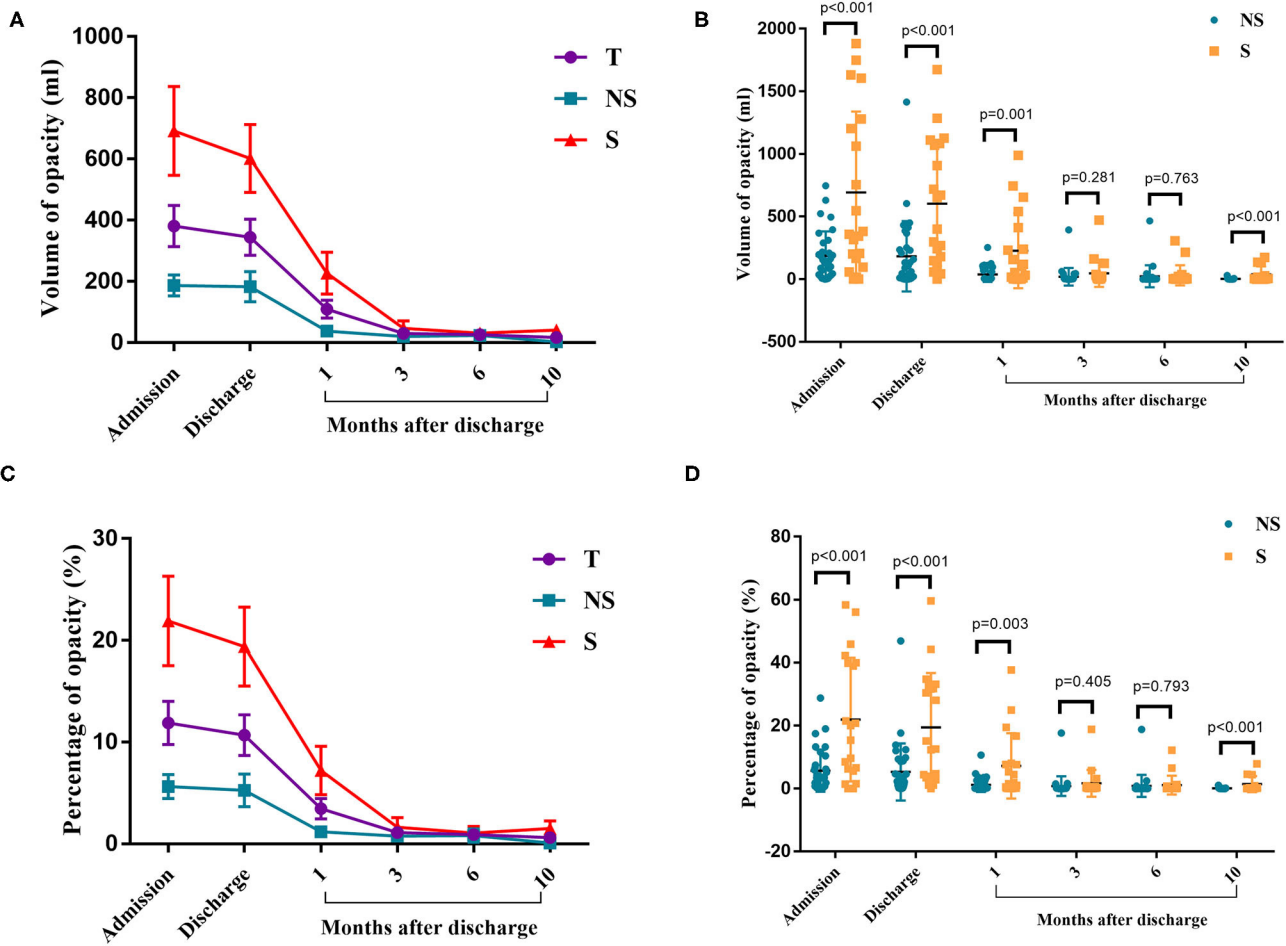
Chest CT results. **(A)** The changes in the volume of opacity for each time point. **(B)** Volume of opacity in the severe and non-severe groups. **(C)** The changes in the percentage of opacity for each time point. **(D)** The percentage of opacity in the severe and non-severe groups. T, total; NS, non-severe; S, severe.

**Figure 3:**
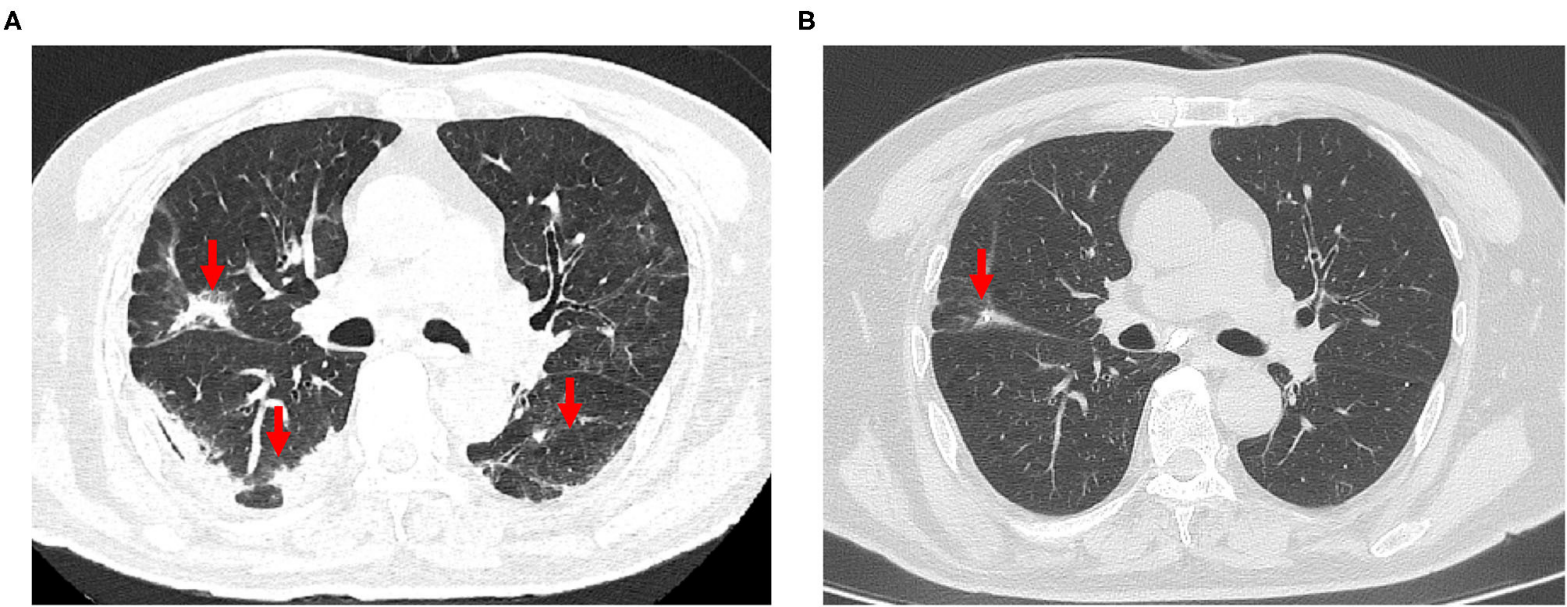
**(A)** The chest CT scan of a 79-year-old man with severe COVID-19 on admission, revealing diffuse ground-glass opacity and consolidation in the lungs. **(B)** Reexamination of chest CT at 10 months after discharge, showing that the ground glass opacities and consolidation had completely disappeared, and mild pulmonary fibrosis were present.

CT imaging data at the time of admission and discharge and at 1, 3, 6, and 10 months after discharge were collected for 52 patients (32 in the non-severe group and 20 in the severe group) and analyzed. The analyzed indexes included lung opacity volume, the percentage of opacity volume accounting for the whole-lung volume (opacity percentage) and the presence of fibrotic lesions. The results were as follows: (1) the lung opacity volume and opacity percentage both gradually decreased over time (Figures 2A,C). The lung opacity volume decreased by 91.9% on average at 3 months after discharge relative to the value at admission (89.1% in the non-severe group and 93.2% in the severe group) and decreased by 95.5% on average at 10 months after discharge relative to the value at admission (98.6% in the non-severe group, 94.5% in the severe group). (2) 10 months after discharge, a total of six patients (18.8%) had residual fibrotic lesions revealed by CT, including five patients in the severe group (38.5%) and one patient in the non-severe group (5.3%), a significant difference was observed between groups (*P* < 0.001). Figure 3A shows a CT image for a severe case at admission, in which a diffuse ground glass opacity accompanied by lung consolidation was observed. Figure 3B shows a CT image of the same patient at 10 months after discharge. The lung opacities had essentially disappeared, whereas several fibrotic lesions remained. (3) At admission, discharge, and 1 month after discharge, the opacity volume and opacity percentage in the severe group were all significantly higher than those in the non-severe group (*P* < 0.05, Figures 2B,D). There were no differences in the opacity volume and opacity percentage between groups at 3 and 6 months after discharge (Figures 2B,D). (4) The volume and percentage of lung opacity showed statistical differences again between the two groups 10 months after discharge. At 10 months after discharge, 12.5% (4/32) of patients still had lung opacity >1% (1.0, 4.4, 4.5, 7.8%, respectively).

### CPET Results

**Figure 4:**
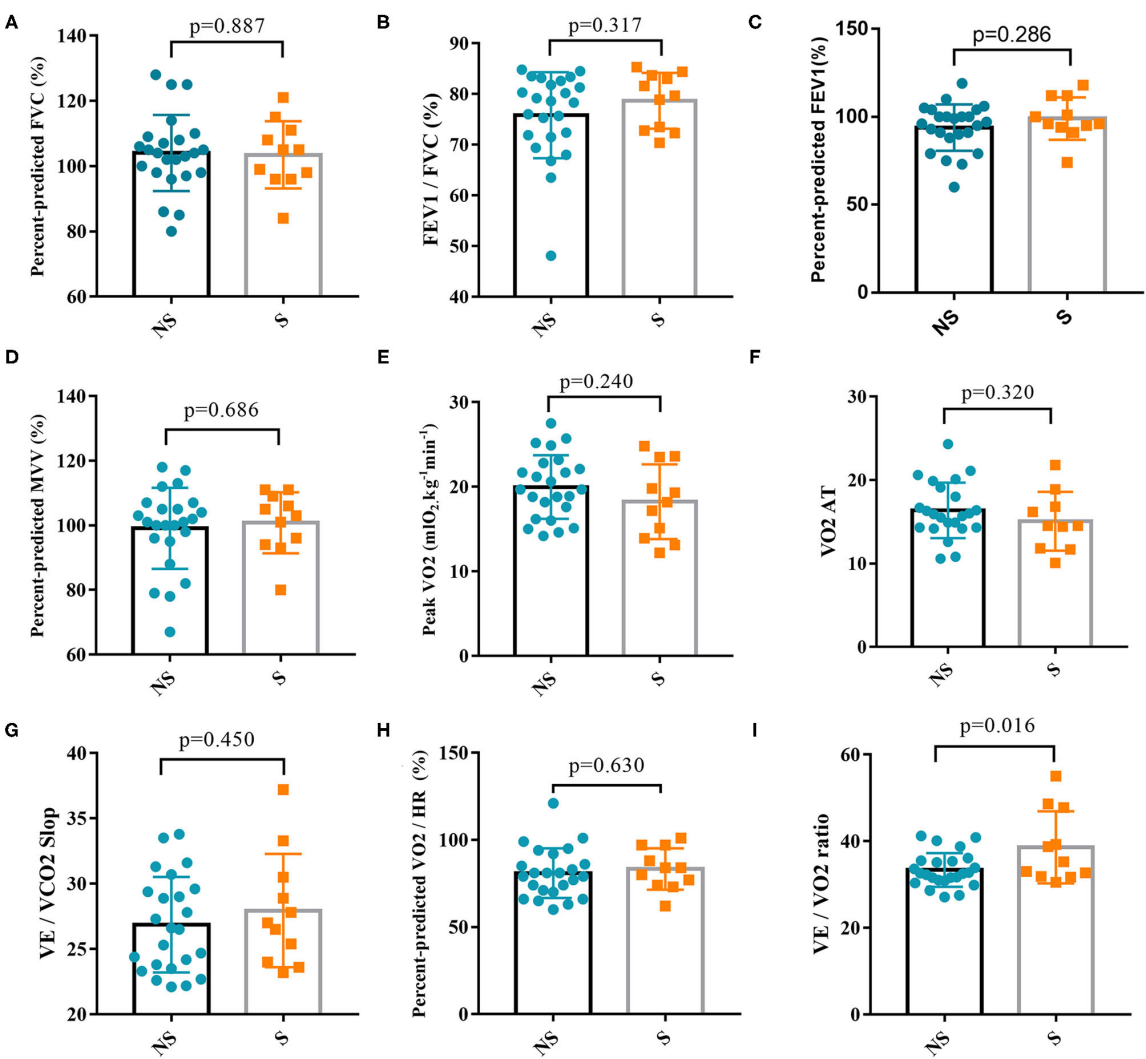
Comparison of cardiopulmonary exercise test parameters between the severe group and non-severe group. **(A)** Percent-predicted FVC between the two groups. **(B)** FEV1/FVC between the two groups. **(C)** Percent-predicted FEV1 between the two groups. **(D)** Percent-predicted MVV between the two groups. **(E)** Peak VO2 between the two groups. **(F)** VO2 AT between the two groups. **(G)** VE/VCO2 Slop between the two groups. **(H)** Percent-predicted VO2/HR between the two groups. **(I)** VE/VO2 ratio between the two groups. FVC, forced vital capacity; FEV1, forced expiratory volume in one second; MVV, maximal voluntary ventilation; Peak VO2, peak oxygen uptake; AT, anaerobic threshold; VE, minute ventilation; HR, heart rate; VCO2, carbon dioxide production; NS, non-severe; S, severe.

Thirty-five patients (24 patients in the non-severe group and 11 patients in the severe group) completed CPET at 6 months after discharge. Regarding complications, there were three cases of hypertension (two cases in the non-severe group and one case in the severe group), one case of diabetes (in the severe group), one case of coronary heart disease (in the non-severe group), and one case of a history of tuberculosis (in the non-severe group). The following indexes were measured and analyzed: FVC%Pred, FEV1/FVC%Pred, FEV1%Pred, MVV%Pred, peak VO_2_, VO_2_ AT, VE/VCO_2_ slop, and VO_2_/HR. We first determined whether the above indexes met the reference values and then stratified the patients on the basis of corresponding indexes (Table 2). All patients had normal FVC%Pred, 22.9% of patients had FEV1/FVC%Pred <92%, 17.1% of patients had FEV1%Pred < 80%, and 9.6% of patients had MVV%Pred < 80%. In 60.0% of patients, the peak VO_2_ was < 20 mlO_2_/kg/min. In 14.3% of patients, the peak VO_2_ was 10–15 mlO_2_/kg/min. A total of 20% of patients had a VO_2_ AT < 14 mlO_2_/kg/min. Three patients had a VO_2_ AT of 8–11 mlO_2_/kg/min, and one of them, a 72-year-old woman, had coronary heart disease and was in the severe group. The other two patients were a 70-year-old man and a 57-year-old woman. A total of 22.9% of patients had a >30% VE/VCO_2_ slope, and 45.7% of patients had O_2_/HR%Pred < 80%. There were no significant differences in all the above parameters between groups (*P* > 0.05) (Figure 4). However, VE/VO_2_ was significantly higher in the severe group (38.60 ± 2.50) than the non-severe group (33.38 ± 0.80) (*P* = 0.016). The follow-up was continued for 6 months after CPET. No new patients with COVID-19 and no recurrence of SARS-CoV-2 RNA positivity were found, thus indicating that CPET is safe for patients with COVID-19 6 months after discharge.

**Table 2:** Result of CPET (Cardiopulmonary exercise testing).

	**Total (*n* = 35)**	**Non-severe (*n* = 24)**	**Severe (*n* = 11)**	***P***
FVC (% predicted) ≥80%			/
Yes, *n* (%)	35 (100%)	24 (100%)	11 (100%)
No, *n* (%)	0 (0%)	0 (0%)	0 (0%)
FEV1/FVC (% predicted) ≥92%			0.387
Yes, *n* (%)	27 (77.1%)	17 (70.8%)	10 (90.9%)
No, *n* (%)	8 (22.9%)	7 (29.2%)	1 (9.1%)
FEV1 (% predicted)			0.399
≥80%, *n* (%)	29 (82.9%)	19 (79.2%)	10 (90.9%)
50–80%, *n* (%)	6 (17.1%)	5 (20.8%)	1 (9.1%)
30–50%, *n* (%)	0 (0%)	0 (0%)	0 (0%)
<30%, *n* (%)	0 (0%)	0 (0%)	0 (0%)
MVV (% predicted) ≥80%			0.536
Yes, *n* (%)	32 (91.4%)	21 (87.5%)	11 (100%)
No, *n* (%)	3 (8.6%)	3 (12.5%)	0 (0%)
Peak VO2 (% predicted) ≥80%			1.000
Yes, *n* (%)	6 (17.1%)	4 (16.7%)	2 (18.2%)
No, *n* (%)	29 (82.9%)	20 (83.3%)	9 (81.8%)
Peak VO2 (mlO2 kg^−1^ min^−1^)			0.162
>20, *n* (%)	14 (40.0%)	11 (45.8%)	3 (27.3%)
15–20, *n* (%)	16 (45.7%)	11 (45.8%)	5(45.4%)
10–15, *n* (%)	5 (14.3%)	2(8.3%)	3 (27.3%)
<10, *n* (%)	0 (0%)	0 (0%)	0 (0%)
VO2 AT (mlO2 kg^−1^ min^−1^)			0.139
>14, *n* (%),	28 (80.0%)	21 (87.5%)	7 (63.6%)
11–14, *n* (%)	4 (11.4%)	1 (4.2%)	3 (27.3%)
8–11, *n* (%)	3 (8.6%)	2 (8.3%)	1 (9.1%)
<8, *n* (%)	0 (0%)	0 (0%)	0 (0 %)
VE/VCO2 ≤ 30%			0.685
Yes, *n* (%)	27 (77.1%)	19 (79.2%)	8 (72.7%)
No, *n* (%)	8 (22.9%)	5 (20.8%)	3 (27.3%)
VO2/HR (% predicted) ≥80%			0.493
Yes, *n* (%)	19 (54.3%)	12 (50%)	7 (63.6%)
No, *n* (%)	16 (45.7%)	12 (50%)	4 (36.4%)

*Data are expressed as n (%) of participants. FVC, forced vital capacity; FEV1, forced expiratory volume in one second; MVV, maximal voluntary ventilation; Peak VO2, peak oxygen uptake; AT, anaerobic threshold; VE, minute ventilation; HR, heart rate; VCO2, carbon dioxide production*.

## Discussion

Studies investigating virus-specific IgG and IgM in the acute phase of SARS-CoV-2 infection have found that asymptomatic patients often have weaker immune responses to SARS-CoV-2 and that IgG decreases in the early stage of infection (8). It is well-known that IgM provides the first line of defense against viral infection (9). The majority of COVID-19 patients in our study developed symptoms in January 2020, when there was no method to detect SARS-CoV-2 antibodies. So the antibody data at the initial stage of infection was missing. According to the available data, IgM declines rapidly, and over half of patients with IgM turned negative 12 months after discharge. IgG is the most important indicator in the middle and late stages of infection. Most long-term studies have found that SARS-CoV-1- and MERS-CoV-specific IgG levels gradually decline over time (usually in a follow-up for at least 1 year). Some studies have found that IgG can be detected 3 years after the onset of symptoms. The antibody kinetics are positively associated with the severity of the disease: the more severe the symptoms are, the longer the antibody-detectable duration is (10). Other studies have found that the protective effect may last only 1–2 years after coronavirus infection (11). Chen et al. followed up patients with COVID-19 for 100 days and found that IgG levels dramatically decreased 3–4 months after symptom onset (12). Our results were in agreement with their findings, in that the IgG levels gradually decreased over time. Interestingly, this decline was more pronounced in the first 6 months after discharge, and we found that IgG remained stable during the next 6 months of follow-up. During the 6th to 12th months after discharge, the antibody levels of most patients stayed low but relatively stable. While whether the antibodies can protect patients from reinfection requires further study. The results of our study have many benefits for prevention and control of COVID-19, the most important of which is to help us predict the trend of the COVID-19 pandemic and provide more guidance for the application of vaccines. Because nearly 90% of patients have antibodies lasting more than 1 year, vaccination tends to be recommended. As of 25 March 2021, 462 million doses of COVID-19 vaccine have been administered globally, according to WHO data (1). However, the vaccination ratio is still too low, far from achieving herd immunity. So we recommend expanding the scale of vaccination. In addition, the IgG titer level gradually decreases, so multiple vaccinations may be needed, and the interval between two vaccinations can be tentatively set at 1 year.

CT is an important tool for COVID-19 diagnosis and efficacy evaluation. CT can be used to observe the changes in lung lesions in a timely manner during follow-up and to aid in assessing disease severity, determining intervention approaches, and predicting prognosis (13–15). The most common imaging signs of COVID-19 include ground glass opacities, consolidation, turbidity, and peripheral distribution (16, 17). CT examination of our patients at admission revealed above-typical abnormalities. At admission, during hospitalization and 1 month after discharge, the lung opacity volume and opacity percentage in the severe group were both significantly higher than those in the non-severe group, showing a concordance between lung opacity severity and disease severity: the more severe the disease, the greater the lung opacity volume and opacity percentage. The decrease in pulmonary opacity was most significant within 3 months after discharge. In three patients in the severe group, the opacity percentage remained above 4% at 10 months after discharge, but their CPET results were normal. These findings suggest that a small number of patients with lung opacity require longer times for lesion absorption, although their daily activities may not be affected. We also found that several patients had residual fibrosis, which were more likely to appear in severe cases. Changes in pulmonary fibrosis require longer follow-up times. According to the above results, we suggest that more attention should be paid to the follow-up of pulmonary fibrosis in severe cases.

Symptoms of COVID-19 include fever, cough, dyspnea, and fatigue, which result from the lung and systemic inflammation caused by SARS-CoV-2 (18, 19). Inflammation of the lungs can affect pulmonary blood vessels and lead to ARDS (20, 21). Myocardial injury is also common in patients with COVID-19 and occurs with an incidence of approximately 15.8% (22). This injury usually manifests as acute cardiac injury, ventricular arrhythmia, and hemodynamic instability associated with non-obstructive coronary artery disease (23). Despite clinical recovery, cardiovascular complications are possible (24). The causes of this condition remain unclear. A potential mechanism is the direct myocardial damage mediated by ACE2 (23, 25). Compared with traditional exercise testing, CPET can be used to comprehensively evaluate the pulmonary, cardiovascular, muscular, and cellular oxidation systems and the severity of cardiopulmonary injury, thus aiding in determining rehabilitation and exercise plans, and supporting management strategies for improving patient prognosis. (26–28). No reports have addressed the utility of CPET in patients with COVID-19. However, some researchers have suggested using CPET to monitor pathophysiological changes in patients with COVID-19 to guide treatment (28). To avoid the spread of the disease and cross-infection, we conducted CPET in patients with COVID-19 a half year after patient discharge. Our data showed that the static lung function indexes such as FVC%Pred and MVV%Pred in most patients were within the normal range, but FEV1/FVC%Pred and FEV1%Pred in one-fifth of patients were lower than normal, thus suggesting pulmonary dysfunction. Abnormal peak VO_2_ occurred in 60% of patients, but abnormal VO_2_ AT occurred in only 20% of patients. Comprehensive analysis of the above data, according to Weber classification, indicated that 80% of patients were in class A, with normal cardiac function. Most patients with abnormal peak VO_2_ stopped the test because of leg fatigue rather than chest tightness or shortness of breath. This result is consistent with findings from the most recent study, which has reported that 63% of patients had symptoms of fatigue or muscle weakness 6 months after discharge (29). Three patients were assessed as having severe cardiac insufficiency according to Weber classification. Among them, one patient was a 72-year-old woman with coronary heart disease, one patient was an older man 70 years of age, and the third was a 57-year-old woman without other complications. In nearly half the patients, the VO_2_/HR did not reach the expected values, thus suggesting a relatively poor cardiac reserve. The VE/VO_2_ was higher in the severe group than the non-severe group, thus indicating that the utilization of oxygen in the severe group was lower than that in the non-severe group. These results suggest that SARS-CoV-2 infection may cause abnormal muscle metabolism as well as cardiopulmonary dysfunction. We found that approximately one-fifth of the patients had cardiopulmonary dysfunction. Because of the lack of baseline data before SARS-CoV-2 infection, we cannot determine whether cardiopulmonary dysfunction is related to SARS-CoV-2 infection; therefore, further research is needed.

Some limitations of our study should be considered. First, the sample size of this study was small. Only 248 patients with early-stage COVID-19 visited our treatment center, and nearly one-quarter were enrolled in this study. Patients with different ages and severity levels were included in this study. However, this sample was still considered to be representative. Second, all enrolled patients had only one CPET result, and thus the findings could not be compared dynamically. To avoid SARS-CoV-2 viral transmission and cross-infection to the greatest extent possible, CPET was performed on each patient only once during the follow-up. However, the pandemic has caused infection in a large range of people worldwide, and the previous prognosis of rehabilitated patients was assessed only with imaging data; moreover, the rehabilitation of cardiopulmonary function was unknown. Therefore, these limited data are expected to play an important role in the comprehensive assessment of rehabilitated patients.

In conclusion, our study showed that the IgG antibodies in most patients with COVID-19 can last at least 12 months after discharge. The IgG titers decreased significantly in the first 6 months and remained stable in the following 6 months. The lung lesions of most patients with COVID-19 can be absorbed without sequelae, and a few patients with severe condition are more likely to develop pulmonary fibrosis. Approximately one-fifth of the patients had cardiopulmonary dysfunction 6 months after discharge.

## Data Availability Statement

The raw data supporting the conclusions of this article will be made available by the authors, without undue reservation.

## Ethics Statement

This study was approved by the Medical Ethical Committee of The Second Affiliated Hospital of Chongqing Medical University (reference no. 2020-530) and Chongqing University Three Gorges Hospital (reference no. 2020-57). Written informed consent was obtained from each enrolled patient. The patients/participants provided their written informed consent to participate in this study. Written informed consent was obtained from the individual(s) for the publication of any potentially identifiable images or data included in this article.

## Author Contributions

QS made substantial contributions to the study concept and design and had full access to all of the data in the study and took responsibility for the integrity of the data and the accuracy of the data analysis. KX was in charge of the manuscript draft. KX, BL, and HY made substantial contributions to the data acquisition, analysis, and confirmation of data accuracy. JC, LY, and XJ took responsibility for obtaining written consent from patients, obtaining ethical approval, and collecting samples. ML, ZZ, JiD, HM, and YL were responsible for the analysis and interpretation of images and antibodies. KX, JC, XP, SD, JuD, and YY collected the data. QS, KX, BL, and HY verified the underlying data. All the authors critically revised the manuscript for important intellectual content and gave final approval for the version to be published.

## Conflict of Interest

The authors declare that the research was conducted in the absence of any commercial or financial relationships that could be construed as a potential conflict of interest.
